# The Impact of Migration on Attitudes to Female Genital Cutting and Experiences of Sexual Dysfunction Among Migrant Women with FGC

**DOI:** 10.1007/s11930-018-0139-4

**Published:** 2018-02-23

**Authors:** Sara Johnsdotter

**Affiliations:** Faculty of Health and Society, Malmö University, 205 06 Malmö, Sweden

**Keywords:** Female genital cutting, Female genital mutilation, Migration, Discourse, Sexual dysfunction, Sexual self-image

## Abstract

**Purpose of Review:**

The purpose of this review was to explore current research on the impact of migration on issues related to female genital cutting and sexuality.

**Recent Findings:**

There is growing evidence that migration results in a broad opposition to female genital cutting among concerned migrant groups in western countries. In addition, after migration, affected women live in the midst of a dominant discourse categorizing them as “mutilated” and sexually disfigured. There is also, in contrast to what is shown by most research, a public discourse saying that female genital cutting (FGC) leads to lost capacity to enjoy sex. Concurrently, a vast body of research demonstrates a strong correlation between a negative body image or body shame and sexual dysfunction.

**Summary:**

Care for women with FGC needs to be holistic and, while offering medical care when needed, the health care providers should avoid feeding into self-depreciatory body images and notions about lost ability to enjoy sexual life.

## Introduction

### Background

**Fig. 1:**
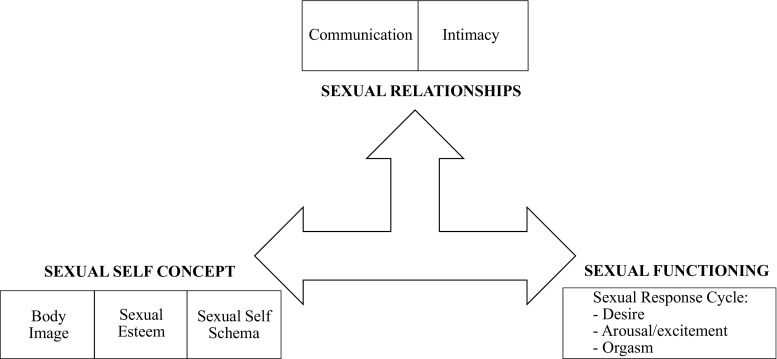
Model for a theoretical overview of sexuality (from Cleary and Hegarty [1])

Sexuality involves a wide range of aspects (Fig. 1), among which interpersonal communication and intimacy can be seen as one axis, and sexual self-concept, body image, sexual esteem, and self-schema another [1]. The “sexual functioning” axis in this model, including desire, arousal/excitement, and orgasm, is partly mirroring the subcategories of “female sexual dysfunctions” in DSM-5 [2]**.**

The research question that guided the search for relevant and current scientific literature was as follows: *What is the impact of migration and acculturation on attitudes to female genital cutting* (*FGC*), *on sexual self-image and experiences of sexual dysfunction among women who have been subjected to FGC?*

The literature review was based on block searches for scientific papers discussing subthemes of the overarching research question. The main area of interest in this literature review was the significance of the sexual self-concept axis.

### Migration and Change of Attitudes Toward FGC

Migration from countries where FGC is practiced to countries where it is not customary, and even illegal, leads to cultural change and declining support of this practice in these groups [3•, 4–8, 9•, 10, 11•, 12–18]. For example, in Sweden, a quantitative study shows that 96% of the Swedish Somalis are opponents of all forms of FGC that includes removal of any tissue [15, 16], similar to what has been found in a quantitative study among Norwegian Somalis [7]. An EU organ, European Institute of Gender Equality, now includes a component to calculate the impact of migration in their formula to estimate girls at potential risk and the total number of women and girls affected in European member states [19].

### Migration and Change of Body Image and Sexual Self-Esteem

In countries where FGC is practiced, it is an often-cited motive that the practice enhances the female body and perfects its genitals [20–24]. Another effect of migration beside changed attitudes toward the practice itself is a process of revaluation among women with FGC regarding their body image and sexual self-esteem, not least in the light of the “mutilation” terminology used in western countries [22••, 23•]. Psychological expectations are crucial for how we experience sex, and self-esteem and body image are at the core of how we see ourselves as sexual beings. Negative body image is clearly related to sexual dysfunction [25–28, 29•, 30, 31, 32]. It can also be noted that in a recently published paper, it is demonstrated that in men, “attitude toward one’s circumcision status is more important than actual circumcision status for men’s body image and sexual functioning” [33•]. Consequently, the ubiquitous public discourse about “mutilation” will have a negative impact on cut women’s body image, sexual self-esteem, and, as an effect, their sexual function [20, 22••, 23•, 34, 35••].

## Studies on Sexual Function Among Cut Women in Western Host Societies

### Quantitative Studies on Sexual Function

Catania et al., in a pioneer study published in 2007 [22••], investigated sexual function among 137 women with female genital mutilation/cutting (FGM/C) living in Italy. Semi-structured interviews and the Female Sexual Function Index (FSFI) were conducted. In one of the substudies (with FSFI), there was a research group including 57 infibulated women and a control group consisting of 57 women without FGM/C (3 Somali and 54 Italian). Catania et al. found that the research group of women with FGM/C actually scored higher in the domains of desire, arousal, and orgasm during sexual intercourse and satisfaction. Regarding lubrication and pain, no significant differences between the groups could be found. Their overall conclusion was that FGM/C per se might not have a negative impact on psychosexual life.

Andersson et al. [36] conducted a case-control study with women with (*n* = 73) and without FGM/C (*n* = 37), using the Sexual Quality of Life-Female (SQOL-F) questionnaire. They found statistically significant difference between those with FGM/C (mainly Somalis) and those without (mainly Nigerian women), with the women who had been subjected to FGM/C having a lower sexual quality of life than the women in the control group.

Chu and Akinsulure-Smith [3•] carried out a computer-assisted self-interviewing study with several instruments, among them FSFI. Sixty-eight women from Gambia, Guinea, Mali, and Sierra Leone, all living in New York, were included. They found no significant differences between cut and uncut women among those who were sexually active, with one exception: women without FGM/C scored significantly better in the arousal domain.

Abdulcadir et al. [37••] conducted a cross-sectional study including a research group of 15 women with and a control group of 15 women without FGM/C in Switzerland. The participants completed several questionnaires, among them FSFI, and went through a pelvic magnetic resonance imaging. The study showed higher overall scores on FSFI among the women without FGM/C; however, there were not any statistically significant differences between the groups regarding the FSFI dimensions of desire, orgasm, and satisfaction. Women with FGM/C reported more dyspareunia.

The divergent outcomes of these studies among concerned migrant groups in western countries are similar to the variety of results in comparable studies conducted in countries of origin. The sprawling results in studies which include the FSFI questionnaire and both research and control groups [38–42] demonstrate the limitations of such studies. The limitations of quantitative studies to measure sexual outcomes have been discussed, among others, by Abdulcadir et al. [37••], Berg and Denison [43], Catania et al. [22••], Johnson-Agbakwu and Warren [35••], and Obermeyer [44, 45].

### Qualitative Studies About Sexuality in Concerned Immigrant Groups

In some qualitative studies, the issues of sexuality, and sexual function and dysfunction, were raised with the interviewees. Among them, there is the mixed-method study by Vloeberghs et al. [46], in which women from Somalia, Sierra Leone, Sudan, Eritrea, and Ethiopia were included, all of them living in the Netherlands. The interviews were based on four different questionnaires, however not the FSFI. A question about the first sexual encounter was asked, and scattered statements about sexuality were reported in their article, mainly how views of FGC and sexuality had changed due to migration and their living in a new cultural context. Some women also reported that sex was painful and that they avoided sexual contact. The authors pose as a key message of their study that “In a number of cases migration to a Western country may result in chronic psychosocial and sexual problems” [p. 692].

Villani et al. [47] conducted in-depth interviews in Switzerland with seven Somali women and one woman from the Ivory Coast, all with FGC. They reported many problems associated with sexuality. However, just as quantitative studies have limitations, qualitative studies have their own. One such limitation may be about how interviewees are being coached into describing their sexual experiences in certain ways. For example, in this study, interviewees were probed to describe their sexual experience in terms of problems and suffering: “the women received a list of possible problems during sexual activities from which they could select multiple options” [p. 6], and, in a next step, they were encouraged to see a link between their problems and the fact that they were cut: “Although the women did not initially make the connection between infibulation and their health and sexuality, when prompted and given examples, the women related the two” [p. 8].

Connor et al. [4] discuss sexual function in a qualitative study with 30 Somali women in the USA. They found some sexual problems, such as pain due to the small opening following infibulation, but their study also demonstrated the complexity involved in researching sexual experiences cross-culturally: traditional Somali views of sexuality are embedded in religious and cultural frameworks that are different from what is seen as “normal” and putatively scientifically based in western countries [4; see also 48, 49].

Other qualitative studies focus not so much on sexual function, but on how FGC relates to traditional notions about sexual morality, and what implications follow from defibulation or upbringing young girls without FGC in western countries [11•, 50–52].

## Discussion

### The Western Understanding of Sexual Function Has an Impact

In western countries, the dominant view of sexual function and dysfunction focuses extensively on the genitals [44, 53–57]. Consequently, female sexual dysfunction in women, as described in DSM-5 [2], is primarily about disorders including the “behavior” of genital parts [57]. This model builds on a western, culture-specific understanding of sexuality and sexual function [22••, 44, 45, 53, 54, 58]. The bulk of research questions and survey instruments to investigate into sexuality after FGC have been developed in a western context, and it is a moot point how well they capture other culture-specific ways of framing and experiencing sex [59]. Further, the instruments used today to measure “sexual function” in women with FGC, with their research focus on aspects such as arousal and lubrication, do not address the issue of how a stigmatizing discourse may have an actual impact also on physiological processes.

In much of the western public anti-FGM discourse, and also in the WHO classification of different types, it is stated that there can be a “total removal of the clitoris.” This is a misrepresentation, since at even the most extensive forms of FGC, the cutting involves the external tip of the clitoris, while its inner structures remain intact [22••, 37••]. This has been solidly demonstrated in a study where the clitorises in cut and uncut women were studied using magnetic resonance imaging, MRI [37••].

In the wake of this narrow understanding of sexual fulfillment and confusion regarding what can be anatomically removed when FGC is performed, it is not surprising to find expectations that FGC is detrimental to sexual health. However, if FGC would be as harmful to all or most cut women as is often claimed in the public discourse, we would expect much larger differences between women with and without FGC regarding sexual dysfunction. In reality, most studies on sexual function show small differences (albeit statistically significant) in sexual function between women with FGC and those without.

As demonstrated by the compiled body of research, FGC per se may lead to adverse sexual function in some women. However, there is reason to give attention to the fact that also anti-FGM campaigning may lead to sexual dysfunction in cut women, especially in western societies where the discourse is pervasive. Schultz and Lien [60] conducted a study among immigrants with FGC in Norway, investigating sociocultural factors that may prevent that FGC is experienced as a trauma when it is performed on a girl child. They highlight that the context changes after migration: “Converting to a new system of belief and knowledge will force a shift in attitude, from seeing oneself as a clean and honorable woman, perhaps without a clitoris and infibulated, to seeing oneself as a mutilated woman and/or abused child, robbed of her sexuality and injured for life” (p. 216). The “mutilation” discourse results in feelings of loss of identity, of defective femininity, and of sexual disfigurement [20, 22••, 23•, 34, 58, 61], which have a direct negative impact on sexual health. These feelings of being inadequate as regards femininity among women and girls with FGC may be reinforced by the fact that research shows that a growing number of men in concerned immigrant communities are opponents of FGC [6, 7, 15, 16, 18, 61–63] and prefer to marry women who have not been cut [6, 7, 63]. This situation may give rise to a double burden for unmarried women with FGC: they are being categorized as “mutilated” and sexually deprived by public discourse in their host societies, while concurrently they may feel rejected as coveted women among men in their local communities.

### Increasing Demand for Reconstructive Clitoral Surgery

The increasing demand for reconstructive clitoral surgery, and the motives for wanting the operation, is illustrative of how discourse may negatively affect cut women’s body image and sexual self-esteem: in a prospective study including almost 3000 women who had gone through reconstructive clitoral surgery in France, and among whom 861 women attended the 1-year follow-up, 99% stated that their motive for the surgery had been “identity recovery” and 81% had hoped for an “improved sex life” [64]. In a systematic review of the evidence of the surgery in cut women, the authors conclude: “Current advertising campaigns are generating a considerable demand for clitoral reconstruction, despite the absence of conclusive evidence regarding its benefits or absence of harm” [65, p. 96] and in the UK guidelines for obstetricians and gynecologists, they advise against the operation since “current evidence suggests unacceptable complication rates without conclusive evidence of benefit” [66, p. 4]. Given that FGC in traditional contexts is considered to enhance femininity, one can draw the conclusion that the dominant discourse in anti-FGM campaigning contributes considerably to the growing demand for reconstructive clitoral surgery [35••]. In a review of outcomes after surgical interventions after FGC, the authors found differences in motivations for clitoral reconstruction: “some indication that specified reasons among women residing in Western countries and those in African countries are distinctive, with women in the West more often stating restoration of identity and aesthetic improvement as motivations” [67, p. 985]. Yet, it can be noted that many women who have gone through the surgery report that they benefitted from it [64, 67, 68].

### Implications for Care

A review of the scientific literature shows that the narrow western definition of sexual dysfunction (as described in the DSM-5 [2]) is dominant, especially in the medical field. However, some researchers, including physicians [e.g., 22••, 35••, 69•], insist that a broader framework of sexual health be used in the care of women with FGC. It has also been argued that in western countries, cut women “undergo a sort of ‘mental/psychological’ infibulation which could result in iatrogenic sexual dysfunction” [22••, see also 23• and 34] and that they, perhaps especially relevant for adolescents, “may acquire a pathological view of their bodies/sexual function when they live in western cultures that stigmatize FGC” [35••, p. 28]. As a result, there is a call for sexual counseling for women with FGC that takes advantage of a wider arsenal of approaches in care settings. Elements suggested to be included in training of health care professionals who meet women with FGM, beside the medical care such as defibulation, clitoral repair, or other medical interventions, include the following: psychosexual counseling that goes beyond surgical intervention [35••, 37••, 65•, 67, 70, 71•, 72], professional attitudes that are not based on emotional distress [69•] but a non-judgmental and open mind [37••, 73], cultural skills in discussing sexuality [63, 74], and multidisciplinary care teams and holistic care [23•, 70, 71•, 72•, 75••]. In addition, it has been suggested that correct knowledge about anatomy, physiology, and sexuality of women with FGM is important not only for women with FGC [35••, 69•, 71•], but also for western health care providers—for example, correct notions about the size of the clitoris and other tissue of importance for sexual pleasure in women with FGC [35••, 37••, 69•]. In an overview surveying which health care approaches to women with FGM are evidence based [76], the authors found gaps regarding research on various approaches to caring for cut women’s sexual concerns, and thus, future studies are needed. Johnson-Agbakwu and Warren [35••] emphasize the necessity to address all the complex influences on sexual function in research and care. Abdulcadir et al. suggest “a comprehensive, evidence-based approach that does not contribute to stigmatization of women and girls living with FGM is needed to provide optimal care” [65, p. 96]. It is a call for a holistic and sensitive care for women with FGC, an approach that does not evoke feelings of loss of femininity or expectations of sexual dysfunction.

## Conclusions

There is evidence that migration leads to revaluation of the practices involving female genital cutting, resulting in growing opposition to these practices in concerned migrant groups. Concurrently, when migrant women from FGC-practicing countries adapt to a new discourse involving notions about “mutilation,” they risk impaired sexual health due to a worsened body self-image and lowered sexual self-esteem. The compiled body of research on FGC and sexual function shows that most women can have pleasurable sex lives, while some of them need care for negative consequences of the FGC procedure. Consequently, the care system needs to be prepared to deal with women who suffer from medical consequences of the procedure while also being prepared to encourage women who mainly feel “incomplete” and “mutilated” due to the dominant discourse in anti-FGM campaigning. A holistic and multidisciplinary care would cater for all women concerned.

## References

[1] Cleary V, Hegarty J (2011). Understanding sexuality in women with gynaecological cancer. Eur J Oncol Nurs.

[2] American Psychiatric Association (2013). DSM-5. Diagnostic and statistical manual of mental disorders.

[3] Chu T, Akinsulure-Smith AM (2016). Health outcomes and attitudes toward female genital cutting in a community-based sample of West African immigrant women from high-prevalence countries in New York City. J Aggress Maltreat Trauma.

[4] Connor JJ, Hunt S, Finsaas M, Ciesinski A, Ahmed A, Robinson BBE (2016). Sexual health care, sexual behaviors and functioning, and female genital cutting: perspectives from Somali women living in the United States. J Sex Res.

[5] Farina P, Ortensi LE, Menonna A (2016). Estimating the number of foreign women with female genital mutilation/cutting in Italy. Eur J Publ Health.

[6] Gele AA, Kumar B, Hjelde KH, Sundby J (2012). Attitudes toward female circumcision among Somali immigrants in Oslo: a qualitative study. Int J Women's Health.

[7] Gele AA, Johansen EB, Sundby J (2012). When female circumcision comes to the West: attitudes toward the practice among Somali immigrants in Oslo. BMC Public Health.

[8] Gele AA, Sagbakken M, Kumar B (2015). Is female circumcision evolving or dissolving in Norway? A qualitative study on attitudes toward the practice among young Somalis in the Oslo area. Int J Women's Health.

[9] Johnsdotter S, Essén B (2016). Cultural change after migration: circumcision of girls in Western migrant communities. Best Pract Res Clin Obstet Gynaecol.

[10] Johnsdotter S, Mestre i, Mestre RM (2017). ‘Female genital mutilation’ in Europe: public discourse versus empirical evidence. Int J Law Crime Justice.

[11] Koukoui S, Hassan G, Guzder J (2017). The mothering experience of women with FGM/C raising ‘uncut’ daughters, in Ivory Coast and in Canada. Reprod Health.

[12] Ortensi LE, Farina P, Menonna A (2015). Improving estimates of the prevalence of female genital mutilation/cutting among migrants in Western countries. Demogr Res.

[13] Vogt S, Efferson C, Fehr E (2017). The risk of female genital cutting in Europe: comparing immigrant attitudes toward uncut girls with attitudes in a practicing country. SSM – Population Health.

[14] Wade L. Clitoridectomy, female genital cutting practices, and law. The Wiley Blackwell Encyclopedia of Gender and Sexuality Studies, 2016;1–6. 10.1002/9781118663219.wbegss269.

[15] Wahlberg A, Johnsdotter S, Selling KE, Källestål C, Essén B (2017). Factors associated with the support of pricking (female genital cutting type IV) among Somali immigrants—a cross-sectional study in Sweden. Reprod Health.

[16] Wahlberg A, Johnsdotter S, Selling KE, Källestål C, Essén B (2017). Baseline data from a planned RCT on attitudes to female genital cutting after migration: when are interventions justified?. BMJ Open.

[17] Ziyada MM, Norberg-Schulz M, Johansen REB (2016). Estimating the magnitude of female genital mutilation/cutting in Norway: an extrapolation model. BMC Public Health.

[18] Johnson-Agbakwu CE, Helm T, Killawi A, Padela AI (2014). Perceptions of obstetrical interventions and female genital cutting: insights of men in a Somali refugee community. Ethnicity & Health.

[19] European Institute for Gender Equality (2015). Estimation of girls at risk of female genital mutilation in the European Union.

[20] Abdi R (2012). Carving culture: creating identity through female genital cutting. Durham Anthropol J.

[21] Boddy JP (2007). Civilizing women: British crusades in colonial Sudan.

[22] Catania L, Abdulcadir O, Puppo V, Verde JB, Abdulcadir J, Abdulcadir D (2007). Pleasure and orgasm in women with female genital mutilation/cutting (FGM/C). J Sex Med.

[23] Villani M (2017). Reparative approaches in medicine and the different meanings of “reparation” for women with FGM/C in a migratory context. Diversity and Equality in Health and Care.

[24] The Public Policy Advisory Network on Female Genital Surgeries in Africa (2012). Seven things to know about female genital surgeries in Africa. Hastings Cent Rep.

[25] Barzoki MH, Kontula O, Mokhtariaraghi H, Mahboubishariatpanahi N (2017). Dual contradictory effects of self-objectification on sexual satisfaction. Sex Cult.

[26] Claudat K, Warren CS (2014). Self-objectification, body self-consciousness during sexual activities, and sexual satisfaction in college women. Body Image.

[27] Herbenick D, Reece M (2010). Outcomes assessment: development and validation of the female genital self-image scale. J Sex Med.

[28] Herbenick D, Schick V, Reece M, Sanders S, Dodge B, Fortenberry JD (2011). The Female Genital Self-Image Scale (FGSIS): results from a nationally representative probability sample of women in the United States. J Sex Med.

[29] Quinn-Nilas C, Benson L, Milhausen RR, Buchholz AC, Goncalves M (2016). The relationship between body image and domains of sexual functioning among heterosexual, emerging adult women. Sex Med.

[30] Schick VR, Calabrese SK, Rima BN, Zucker AN (2010). Genital appearance dissatisfaction: implications for women’s genital image self-consciousness, sexual esteem, sexual satisfaction, and sexual risk. Psychol Women Q.

[31] Vencill JA, Tebbe EA, Garos S (2015). It’s not the size of the boat or the motion of the ocean: the role of self-objectification, appearance anxiety, and depression in female sexual functioning. Psychol Women Q.

[32] Woertman L, Van den Brink F (2012). Body image and female sexual functioning and behavior: a review. J Sex Res.

[33] • Bossio JA, Pukall CF. Attitude toward one’s circumcision status is more important than actual circumcision status for men’s body image and sexual functioning. Arch Sex Behav 2017. 10.1007/s10508-017-1064-8. **This study points at the importance of attitude to own circumcision for body image and sexual functioning.** 10.1007/s10508-017-1064-8 28894958

[34] Malmström MF (2013). The production of sexual mutilation among Muslim women in Cairo. Global Discourse.

[35] Johnson-Agbakwu C, Warren N (2017). Interventions to address sexual function in women affected by female genital cutting: a scoping review. Curr Sex Health Rep.

[36] Andersson SHA, Rymer J, Joyce DW, Momoh C, Gayle CM (2012). Sexual quality of life in women who have undergone female genital mutilation: a case-control study. BJOG Int J Obstet Gynaecol.

[37] Abdulcadir J, Botsikas D, Bolmont M, Bilancioni A, Djema DA, Demicheli FB, Yaron M, Petignat P (2016). Sexual anatomy and function in women with and without genital mutilation: a cross-sectional study. J Sex Med.

[38] Anis TH, Aboul Gheit S, Awad HH, Saied HS (2012). Effects of female genital cutting on the sexual function of Egyptian women. A cross-sectional study. J Sex Med.

[39] Biglu MH, Farnam A, Abotalebi P, Biglu S, Ghavami M (2016). Effect of female genital mutilation/cutting on sexual functions. Sex Reprod Healthc.

[40] Ibrahim ZM, Ahmed MR, Mostafa RM (2012). Psychosexual impact of female genital mutilation/cutting among Egyptian women. Human Androl.

[41] Alsibiani SA, Rouzi AA (2010). Sexual function in women with female genital mutilation. Fertil Steril.

[42] Ismail SA, Abbas AM, Habib D, Morsy H, Saleh MA, Bahloul M (2017). Effect of female genital mutilation/cutting; types I and II on sexual function: case-controlled study. Reprod Health.

[43] Berg RC, Denison E (2012). Does female genital mutilation/cutting (FGM/C) affect women’s sexual functioning? A systematic review of the sexual consequences of FGM/C. Sex Res Soc Policy.

[44] Obermeyer CM (2003). The health consequences of female circumcision: science, advocacy, and standards of evidence. Med Anthropol Q.

[45] Obermeyer CM (2005). The consequences of female circumcision for health and sexuality: an update on the evidence. Cult Health Sex.

[46] Vloeberghs E, Van der Kwaak A, Knipscheer J, van den Muijsenbergh M (2012). Coping and chronic psychosocial consequences of female genital mutilation in the Netherlands. Ethn Health.

[47] Villani M, Griffin JL, Bodenmann P (2016). In their own words: the health and sexuality of immigrant women with infibulation living in Switzerland. Soc Sci.

[48] Bell K (2005). Genital cutting and Western discourses on sexuality. Med Anthropol Q.

[49] Villani M (2009). From the ‘maturity’ of a woman to surgery: conditions for clitoris repair. Theol Sex.

[50] Johansen REB (2017). Undoing female genital cutting: perceptions and experiences of infibulation, defibulation and virginity among Somali and Sudanese migrants in Norway. Cult Health Sex.

[51] Johansen REB (2017). Virility, pleasure and female genital mutilation/cutting. A qualitative study of perceptions and experiences of medicalized defibulation among Somali and Sudanese migrants in Norway. Reprod Health.

[52] Villani M, Bodenmann P (2017). FGM in Switzerland: between legality and loyalty in the transmission of a traditional practice. Health Sociol Rev.

[53] Esho T, Enzlin P, Van Wolputte S, Temmerman M (2010). Female genital cutting and sexual function: in search of an alternate theoretical model. African Identities.

[54] Obermeyer CM (1999). Female genital surgeries: the known, the unknown, and the unknowable. Med Anthropol Q.

[55] Pronier C, Monk-Turner E (2014). Factors shaping women’s sexual satisfaction: a comparison of medical and social models. J Gend Stud.

[56] Richters J (2009). Bodies, pleasure and displeasure. Cult Health Sex.

[57] Tiefer L (2009). Sex is not a natural act and other essays.

[58] Johnsdotter S, Essén B. Culture and sexual scripts in and out of Africa: understanding FGC in relation to sexuality. Invited paper presented at Management of Women with FGM/C: 1st International Consultation. Paris University/Sorbonne, France, January 27–28, 2015.

[59] Hull TH (2008). Sexual pleasure and wellbeing. Int J Sex Health.

[60] Schultz JH, Lien IL (2014). Cultural protection against traumatic stress: traditional support of children exposed to the ritual of female genital cutting. Int J Women’s Health.

[61] Johnsdotter S, Moussa K, Carlbom A, Aregai R, Essén B (2009). ‘Never my daughters’: a qualitative study regarding attitude change toward female genital cutting among Ethiopian and Eritrean families in Sweden. Health Care for Women Int.

[62] Varol N, Turkmani S, Black K, Hall J, Dawson A (2015). The role of men in abandonment of female genital mutilation: a systematic review. BMC Public Health.

[63] Wahlberg A. Continuity or change? Improved understanding of attitudes towards female genital cutting after migration from Somalia to Sweden. Doct diss, Uppsala University, 2018.

[64] Foldès P, Cuzin B, Andro A (2012). Reconstructive surgery after female genital mutilation: a prospective cohort study. Lancet.

[65] Abdulcadir J, Rodriguez MI, Say L (2015). A systematic review of the evidence on clitoral reconstruction after female genital mutilation/cutting. Int J Gynecol Obstet.

[66] Royal College of Obstetricians and Gynaecologists. Female genital mutilation and its management—Green-top Guideline No. 53 [Internet], 2015. [https://www.rcog.org.uk/globalassets/documents/guidelines/gtg-53-fgm.pdf]. Accessed 20 November 2017.

[67] Berg RC, Taraldsen S, Said MA, Sørbye IK, Vangen S (2017). Reasons for and experiences with surgical interventions for female genital mutilation/cutting (FGM/C): a systematic review. J Sex Med.

[68] Abdulcadir J, Rodriguez MI, Petignat P, Say L (2015). Clitoral reconstruction after female genital mutilation/cutting: case studies. J Sex Med.

[69] Abdulcadir J, Rodriguez MI, Say L (2015). Research gaps in the care of women with female genital mutilation: an analysis. BJOG Int J Obstet Gynaecol.

[70] De Schrijver L, Leye E, Merckx M (2016). A multidisciplinary approach to clitoral reconstruction after female genital mutilation: the crucial role of counselling. Eur J Contracept Reprod Health Care.

[71] Okomo U, Ogugbue M, Inyang E, Meremikwu MM (2017). Sexual counselling for treating or preventing sexual dysfunction in women living with female genital mutilation: a systematic review. Int J Gynecol Obstet.

[72] Hearst AA, Molnar AM (2013). Female genital cutting: an evidence-based approach to clinical management for the primary care physician. Mayo Clin Proc.

[73] Ibe C, Johnson-Agbakwu C (2011). Female genital cutting: addressing the issues of culture and ethics. The Female Patient.

[74] Lazar J, Shipp M, Johnson C (2010). Provider perceptions of sexual desire and dyspareunia among Somali women with female genital cutting. J Sex Med.

[75] Abdulcadir J, Alexander S, Dubuc E, Pallitto C, Petignat P, Say L (2017). Female genital mutilation/cutting: sharing data and experiences to accelerate eradication and improve care. Reprod Health.

[76] Stein K, Hindin MJ, Chou D, Say L (2017). Prioritizing and synthesizing evidence to improve the health care of girls and women living with female genital mutilation: an overview of the process. Int J Gynecol Obstet.

